# Psoriasis: What Is New in Markers of Disease Severity?

**DOI:** 10.3390/medicina60020337

**Published:** 2024-02-18

**Authors:** Mircea Tampa, Madalina Irina Mitran, Cristina Iulia Mitran, Clara Matei, Simona Roxana Georgescu

**Affiliations:** 1 Department of Dermatology, ‘Carol Davila’ University of Medicine and Pharmacy, 020021 Bucharest, Romania; dermatology.mt@gmail.com (M.T.); matei_clara@yahoo.com (C.M.); srg.dermatology@gmail.com (S.R.G.); 2 Department of Dermatology, ‘Victor Babes’ Clinical Hospital for Infectious Diseases, 030303 Bucharest, Romania; 3 Department of Microbiology, ‘Carol Davila’ University of Medicine and Pharmacy, 020021 Bucharest, Romania

**Keywords:** psoriasis, severity, biomarkers

## Abstract

*Introduction*. Psoriasis is a chronic inflammatory skin disease and is the result of the interaction between numerous external and internal factors. Psoriasis presents a wide range of skin manifestations encompassing individual lesions varying from pinpoint to large plaques that can evolve into generalised forms. The lesions mirror the pathophysiological mechanisms involved in psoriasis pathogenesis, such as inflammation, dysregulation of immune response, uncontrolled proliferation of keratinocytes and angiogenesis. In this article, we present the latest advances achieved regarding markers that correlate with psoriasis severity. *Material and method*. We have performed a narrative review on markers of psoriasis severity, including articles published between March 2018–March 2023. *Results*. We have identified four categories of markers: inflammation markers, oxidative stress markers, hormonal markers and cancer-related markers. The main focus was on inflammation biomarkers, including immunomodulatory molecules, haematological parameters, inflammatory cells and costimulatory molecules. *Conclusions*. The analysed data indicate that markers associated with inflammation, oxidative stress and hormones, and cancer-related markers could be useful in assessing the severity of psoriasis. Nevertheless, additional research is required to ascertain the practical importance of these biomarkers in clinical settings.

## 1. Introduction

Psoriasis is a chronic inflammatory skin disease mediated by the immune system. Globally, psoriasis affects 2–3% of individuals, but its prevalence varies by geographical region, reaching up to 10% in some areas and exhibits a dual-peaked distribution, with notable occurrences between 30–39 years and 60–69 years for men, while women experience onset approximately a decade earlier. Psoriasis incidence is equal in males and females [1,2]. Numerous factors that trigger the onset or exacerbation of psoriasis lesions have been identified. These factors can be divided into two main categories, intrinsic and extrinsic factors. The extrinsic factors identified are mechanical stress (Koebner phenomenon), infections (the well-known association between psoriasis and streptococcal infections), vaccination (influenza, BCG, adenovirus), smoking, alcohol consumption and certain drugs (e.g., beta-blockers, lithium, interferons, imiquimod, anti-malarial drugs, etc.). The main intrinsic factors associated with the onset and exacerbation of psoriasis are metabolic syndrome, diabetes mellitus, obesity and psychological stress [3,4].

**Figure 1:**
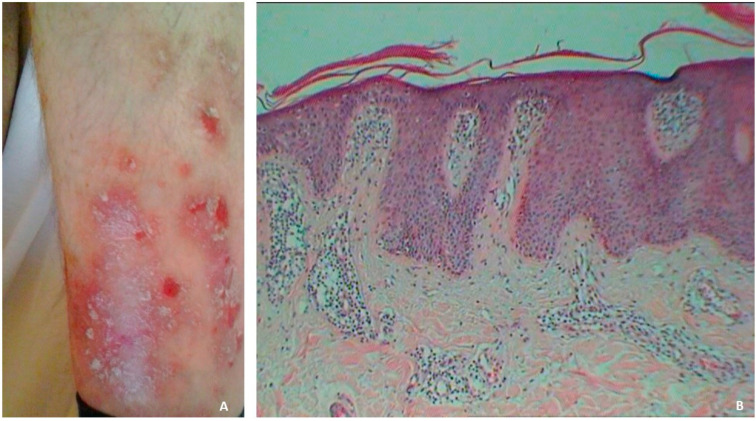
Clinical and histopathological aspects of psoriasis. (**A**). Erythematous plaques covered with silvery-white scales with well-defined edges on the lower limb. (**B**). Parakeratosis, elongation of the rete ridges, enlargement of the dermal papillae, a prominent inflammatory infiltrate in the dermis (HE staining, ×100).

Chronic inflammation, autoimmunity and genetic predisposition seem to be the main actors that orchestrate its pathogenesis [5]. Current evidence suggests that hormonal factors may modulate the course of psoriasis [6]. Moreover, it is presumed that oxidative stresses have a pivotal role in psoriasis pathogenesis [7]. The most frequent form is plaque psoriasis. Psoriasis plaques are erythematous, round oval, well demarcated, of different sizes, isolated or confluent covered by white-silver scales. The scales are lamellar, can be easily removed and become adherent to the indurated plaques in the chronic forms (Figure 1). The lesions can be located anywhere, but most frequently, they involve the extension areas (knees, elbows), scalp and lumbosacral area [8]. Less common forms of psoriasis are guttate, erythrodermic and pustular psoriasis. These forms have distinctive morphologies. Guttate psoriasis is characterised by an eruption consisting of numerous small erythematosquamous papules. In the pustular forms, sterile pustules appear on an erythematous background, and depending on the affected areas, the pustular type is classified as generalised pustular psoriasis, palmoplantar pustulosis or acrodermatitis continua of Hallopeau. Erythroderma is a severe type of psoriasis that can also be considered a complication of the disease, affecting up to 3% of those who suffer from psoriasis. Depending on the location of psoriasis lesions, several particular variants have been described: flexural psoriasis, palmoplantar psoriasis, sebopsoriasis and nail psoriasis [9].

Psoriasis forms range from mild to moderate and severe. The gold standard for the diagnosis is the histopathological examination. Psoriasis is characterised by acanthosis, parakeratosis, microabscesses of Munro in the stratum corneum, elongated rete ridges, elongated dermal papillae, vasodilatation of dermal vessels and a dense inflammatory infiltrate in the dermis (Figure 1) [10].

In recent years, important progress has been achieved in the treatment of psoriasis. Topical therapies remain the mainstay of treatment in mild forms, with few side effects. The use of biological agents represents a particularly important step in the management of moderate and severe psoriasis. The main therapeutic targets are TNF alpha, the p40 subunit of cytokines IL-12 and IL-13, IL-17 and the p19 subunit of IL-23. Biologics are effective and generally well tolerated, but not all patients respond appropriately to therapy. That is why research in this field is ongoing; recent data indicate that blocking the JAK-STAT pathway with small molecules can offer an effective and safe therapeutic option for psoriasis patients [11,12].

There are several scales for assessing the severity of the disease, the most commonly used being the psoriasis area and severity index (PASI) [13]. Recent studies have focused on discovering molecules that correlate with the severity of psoriasis and may offer new insights into its pathogenesis. The aim of this article is to present the latest advances in this field.

## 2. Materials and Methods

**Figure 2:**
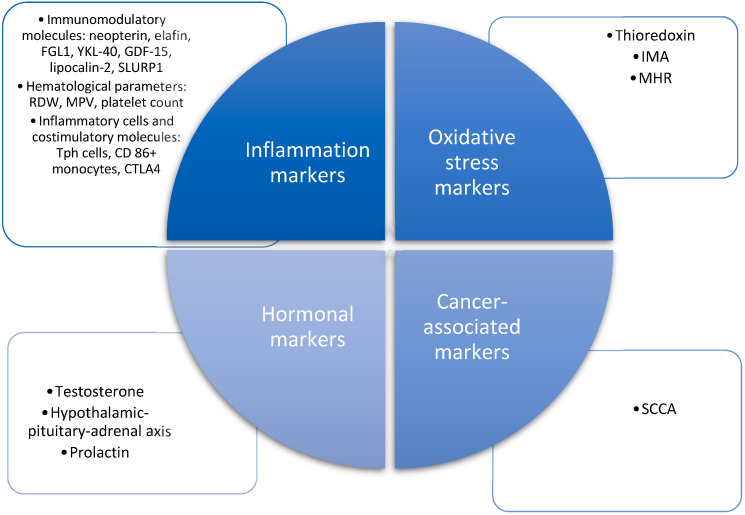
Summary of the markers discussed in this review. FGL1—fibrinogen-like protein I, GDF-15—growth/differentiation factor-15, SLURP1—the secreted Ly6/urokinase-type plasminogen activator receptor-related protein-1, RDW—red cell distribution width, MPV—mean platelet volume, Tph cells—peripheral helper T cells, CTLA4—cytotoxic T-lymphocyte-associated protein 4, IMA—ischemia-modified albumin, MHR—monocyte-to-HDL cholesterol ratio, SCCA—squamous cell carcinoma antigen.

We have performed a narrative review. For the literature research, we searched PubMed and Google Scholar databases using keywords: psoriasis, marker, severity. We searched articles published between March 2018 and March 2023 and focused on four categories of inflammation markers, oxidative stress markers, hormonal markers and cancer-related markers (Figure 2).

## 3. Results

### 3.1. Inflammation-Related Markers

The results of the recent studies analysed in this review are summarised in Table 1, Table 2 and Table 3.

The initiation phase of psoriasis is triggered by external factors such as trauma, chemicals or micro-organisms that lead to the generation of nucleotides within host cells. These nucleotides form a complex when combined with antimicrobial peptides (such as human β-defenin 2, lipocalin 2 and the cathelicidin LL37) produced by activated keratinocytes. Antimicrobial peptides are involved not only in killing pathogenic micro-organisms but also in modulating host inflammatory responses. The skin microbiome seems to play a pivotal role in the pathogenesis of psoriasis. Recent data suggest that micro-organisms, including bacteria (*Staphylococcus aureus*, *Streptococcus pyogenes*), viruses (human papillomavirus, endogenous retroviruses) and fungi (*Malassezia* spp., *Candida albicans*) may exacerbate or initiate psoriasis lesions [25]. Substantial reductions in the richness and diversity of the microbiome were observed in samples from psoriasis lesions compared to those from non-lesional areas and control samples [26]. Antigen-presenting cells, like dendritic cells, promote the proliferation of cutaneous T cells as well as the T cells in the lymph nodes. Following this, dendritic cells produce IFN alpha, subsequently triggering myeloid dendritic cells to secrete IL-23 and TNF. IL-23 promotes the differentiation of T cells into Th1, Th17 and Th22. The released cytokines enhance the synthesis of IL-17 and IL-22 by Th17 and Th22 cells. Notably, IL-17 plays a pivotal role in promoting the generation of TNF, CCL20 and antimicrobial peptides, resulting in an inflammatory skin reaction and increased keratinocyte turnover [27,28]. Based on present knowledge, specific cytokines such as TNF alpha, IL-23 and IL-17 stand out as crucial inflammatory molecules to target. Among them, TNF alpha possesses a more extensive understanding because of its earlier discovery and widespread utilisation as a target for antibody treatment compared to the IL-23/IL-17 axis [28]. Therefore, the hallmark of psoriasis is chronic inflammation, which is mediated by a plethora of more or less known cells and molecules.

#### 3.1.1. Immunomodulatory Molecules

*a. Neopterin*. Neopterin is a compound primarily synthesised by macrophages and monocytes and serves as a marker of immune system activation [29]. Some authors suggest that it can also be seen as a marker of oxidative stress. Elevated levels of neopterin have been shown in infectious diseases, autoimmune conditions and neoplasia [30]. Kemeriz et al. suggest that serum levels of neopterin could represent a promising indicator in assessing the severity of psoriasis. They found that serum neopterin levels were significantly higher among patients with severe psoriasis compared to those with mild-to-moderate psoriasis, and these levels decreased following narrow-band UVB therapy [14]. Similar results have been obtained in the study conducted by Hegazy et al. [31]. Furthermore, it has been demonstrated that urinary neopterin levels decrease following etanercept therapy [32].

*b. Elafin*. Elafin is an epithelial protein that is absent in healthy skin and is secreted by keratinocytes in the presence of IL-1 and TNF-alpha during inflammation [33]. Elafin is considered a molecule with anti-inflammatory properties because it inhibits neutrophil elastase and other proteolytic enzymes. Additionally, it reduces NF-kappa B activity, leading to a decrease in the production of pro-inflammatory cytokines [34].

Elgharib et al. evaluated serum levels of elafin in 26 psoriasis patients and 26 healthy subjects, and they found that serum elafin levels were statistically significantly higher in psoriasis patients. Serum elafin levels correlated with disease severity and inflammatory markers such as erythrocyte sedimentation rate (ESR) and C-reactive protein (CRP) [15]. In a study that focused on the investigation of genes possibly involved in the development of psoriasis, Oestreicher et al. showed increased expression of the gene encoding elafin in psoriatic skin samples [35]. However, Holmannova et al. did not find a positive correlation between serum elafin levels and the severity of psoriasis [34]. Alkemade et al. showed that serum elafin levels decrease following cyclosporine therapy [36].

*c. Fibrinogen-like protein I (FGL1)*. FGL1, a liver-secreted protein, has been demonstrated to act as an immunosuppressive agent in regulating the immune system [37]. Fibrinogen-like protein 1 (FGL1) is considered a major ligand of lymphocyte activation gene-3 (LAG-3) on activated T cells [37]. It appears to be a novel marker that negatively correlates with psoriasis severity; according to a study conducted by Sun et al. in the serum of psoriasis patients, low levels of FGL1 were measured. Additionally, the study highlighted that measuring serum FGL1 levels provides better results in assessing psoriasis severity compared to serum IL-17 levels. Furthermore, a negative correlation was observed between HDL cholesterol levels and FGL1, indicating a possible role of FGL1 in psoriasis forms associated with lipid metabolism disorders [16].

*d. YKL-40*. YKL-40 is a 40 kDa heparin- and chitin-binding glycoprotein secreted by various cells, including chondrocytes, vascular smooth muscle cells and macrophages, and is involved in important processes such as inflammation, angiogenesis and tissue remodelling [38]. Its name is related to three amino acids, tyrosine (Y), lysine (K) and leucine (L), found in the secreted form. Khashaba et al. suggest that YKL-40 could serve as a marker of severity in psoriasis. They conducted a study that included 28 patients with moderate to severe plaque psoriasis and found a positive correlation between the serum levels of YKL-40 and IL-17. Following NB-UVB therapy, a decrease in serum YKL-40 levels was observed, especially in patients with severe psoriasis [17]. The involvement of YKL-40 in the pathogenesis of psoriasis is not fully elucidated. It appears that YKL-40 induces IL-17-mediated neutrophilia, which stimulates inflammatory responses. Additionally, YKL-40 may be involved in endothelial dysfunction that is encountered in some psoriasis patients [17]. However, Baran et al. suggest that YKL-40 is a marker of inflammation in psoriasis rather than a marker that correlates with disease severity [39].

*e. Growth/differentiation factor 15 (GDF-15)*. GDF-15 belongs to the transforming growth factor-β (TGF-β) superfamily. GDF-15 is a cytokine produced in various tissues, its production being particularly elevated in conditions related to mitochondrial stress. Its expression is induced by IL-1beta, TNF-alpha and IL-2. In healthy individuals, GDF-15 has been identified primarily in the placenta and prostate, but low levels have also been detected in the urinary and gastrointestinal tract. GDF-15 can be viewed as a regulator of the immune response, mainly affecting dendritic cells and immune cell migration [40]. Akbari et al. conducted the first study evaluating the role of GDF-15 and its gene expression in peripheral blood mononuclear cells in psoriasis patients [18]. The authors highlighted that serum levels and gene expression of GDF-15 were higher in psoriasis patients compared to the control group and positively correlated with disease severity. They suggested that GDF-15 acts as a β2 integrin-antagonist that traps leukocytes in the endothelium and inhibits their migration to lesional skin, which might explain this finding [18].

Furthermore, a recent study shows that GDF-15 can be used as a marker to detect subclinical cardiovascular disease in patients with moderate to severe psoriasis. A negative correlation has been observed between the level of GDF-15 and inflammation of the aortic wall, and a positive correlation with carotid intima-media thickness, carotid artery plaques, and coronary artery calcium score [41].

*f. Lipocalin-2*. Lipocalin-2 is a novel, 198 amino acid adipocytokine that is also known as neutrophil gelatinase-associated lipocalin. Its main role is to transport small and hydrophobic molecules such as fatty acids, prostaglandins and hormones. However, recently, it has been shown that lipocalin-2 exhibits antibacterial and anti-inflammatory functions [42]. A recent study underlies the role of lipocalin-2 in the pathogenesis of psoriasis and its link with disease severity. The authors have shown that the levels of lipocalin-2 are higher in those with acute forms of psoriasis, such as erythrodermic and pustular psoriasis, compared to those with chronic forms. Also, the authors have highlighted a positive correlation between the level of lipocalin-2 and disease severity using different scales (PASI, body surface area), including those used for nail or pustular psoriasis (nail psoriasis severity index, and pustular severity index) [19]. These results are in line with those obtained by Romani et al. [43] but in contrast to those obtained by Baran et al.; they found no correlation between serum lipocalin-2 levels and disease severity, with serum lipocalin-2 levels not significantly influenced by topical treatment of psoriasis lesions [44].

*g. SLURP1*. The secreted Ly6/urokinase-type plasminogen activator receptor-related protein-1 (SLURP1) is an 88 amino acid peptide that pertains to the leukocyte antigen-6 (Ly6) family [45]. SLURP1 is considered a late marker of epidermal differentiation that plays a role in various processes, such as intracellular signal transduction, immune response activation and cell adhesion [46]. Assaf et al. demonstrated that the intensity of SLURP1 immunohistochemical staining was notably higher in psoriatic lesions compared to unaffected skin. Furthermore, they established a strong correlation between the intensity of SLURP1 immunohistochemical staining and the severity of the disease [20]. Moriwaki et al. used a mouse model of psoriasis induced by imiquimod and observed increased SLURP1 expression in the skin affected by lesions. They proposed that this upregulation was likely mediated through the IL22-STAT3 signalling pathway, suggesting a potential role of SLURP1 in psoriasis development [47].

#### 3.1.2. Hematologic Parameters

*a. Platelet count, b. mean platelet volume (MPV) and c. red cell distribution width (RDW)*. The evaluation of complete blood cell components is easily performed in everyday medical practice, and a recent study suggests that it can provide important data regarding the management of psoriasis patients. Recently, Balevi et al. conducted a study on 45 patients with psoriasis and concluded that RDW might provide a faster result regarding the severity of psoriasis vulgaris compared to measuring PASI values in the initial stages of treatment [48].

A study by Nageen et al. included 120 psoriasis patients, and platelet count, MPV and RDW were determined. The platelet count negatively correlated with disease severity. The authors explain this phenomenon by the fact that numerous platelets are attracted to psoriasis lesions as part of the inflammatory process, leading to a decrease in circulating platelets [21]. Regarding MPV, no correlation with PASI scores was identified; however, a previous study indicated that elevated MPV values correlated with disease severity [49]. Regarding RDW, no positive correlation with PASI was identified [21].

#### 3.1.3. Inflammatory Cells and Costimulatory Molecules

*a. Peripheral helper T cells (Tph cells)*. According to the expression of CXCR3 and chemokine CCR 6, Tph cells are classified into three subgroups: CXCR3+CCR6− Tph1 cells, CXCR3−CCR6− Tph2 cells and CXCR3−CCR6+ Tph17 cells. Tph cells appear to participate in the development of psoriasis lesions. Liu et al. identified an increased number of this subset of cells in psoriasis patients. Additionally, a higher activation status of circulating Tph17 cells was observed. The frequency of Tph17 cells positively correlated with disease severity, which underlies their role in psoriasis pathogenesis [22].

*b. Monocytes and CD86*. Monocytes play an essential role in inflammation and are classified into three subsets based on the expression of CD14 and CD16 markers: CD14++CD16− monocytes (classical monocytes, the majority subset), CD14++CD16+ monocytes (intermediate monocytes) and CD14+CD16++ monocytes (non-classical monocytes). Intermediate and non-classical monocytes are found in increased numbers in patients with disorders where inflammation is the dominant process, such as asthma or rheumatoid arthritis. There is limited literature available regarding their number in psoriasis [50].

Given that an increased number of circulating monocytes correlates with psoriasis severity, recent research has found that elevated CD86 expression on intermediate monocytes is associated with psoriasis severity. The presence of CD86+ CD14+ CD16+ cells in psoriasis lesions is linked to increased keratinocyte proliferation. Phototherapy and biologic therapy downregulate the expression of CD86, suggesting that CD86 could be used as a marker for monitoring treatment response in psoriasis patients [23].

*c. Cytotoxic T lymphocyte antigen-4 (CTLA-4)*. In psoriasis, the initial activation of T cells is triggered by a primary signal from the antigenic peptide presented by major histocompatibility complex (MHC) molecules and a signal resulting from the interaction of costimulatory molecules. The costimulatory signal results from the interaction between CD28, which is located on T cells, with CD8 and CD86, expressed by antigen-presenting cells. CTLA-4, also known as CD152, belongs to the immunoglobulin superfamily and shares structural similarities with CD28. It is specifically present on activated T-helper (Th) cells and functions as a suppressor of the T-cell response. CD28 and CTLA-4 bind the ligands CD80 and CD86 that are found on antigen-presenting cells [51].

Liu et al. investigated the relationship between psoriasis severity and the expression of CTLA-4 and obtained intriguing results. Soluble CTLA-4 did not correlate with the severity of psoriasis. However, membrane CTLA-4 expression in the skin was higher in moderate forms of psoriasis compared to severe forms. Moreover, blocking CTLA-4 function in a mouse model of psoriasis induced by imiquimod was associated with increased epidermal thickness and high amounts of CD3+ T cells [24].

**Table 2:** The link between oxidative stress-related markers and disease severity.

Marker	Correlation with Disease Severity	Reference
TrxR	negative	Kiafar et al. [52]
IMA	positive	Tampa et al. [53]
MHR	positive	Sirin et al. [54]

TrxR—thioredoxin reductase, IMA—ischemia-modified albumin, MHR—monocyte-to-HDL cholesterol ratio.

### 3.2. Oxidative Stress-Related Markers

Current evidence indicates that oxidative stress is a major contributor to the onset of psoriasis. Numerous studies have shown low levels of oxidative stress markers in psoriasis lesions and in the blood or saliva of psoriasis patients in association with low levels of antioxidants, highlighting the imbalance between pro-oxidants and antioxidants [55,56,57,58,59]. There is a close connection between oxidative stress and chronic inflammation; they are two interconnected processes [60,61]. Multiple external factors such as smoking, environmental pollution, physical trauma and micro-organisms can indirectly damage the keratinocytes by triggering an excessive production of reactive oxygen species (ROS). Therefore, the integrity of cell membranes in the skin is altered, the main molecular targets of oxidative stress being lipids, proteins and nucleic acids. Oxidative stress in psoriasis triggers the initiation of numerous signalling pathways (such as NF-κB and MAPK, STAT 3), leading to the activation of Th1 and Th17 cells and the release of a plethora of pro-inflammatory cytokines that contribute to the hyperproliferation of keratinocytes, immune cell infiltration into the skin and alterations in blood vessel permeability [62]. The intensified inflammatory response exacerbates ROS production and diminishes the already compromised antioxidative capacity, resulting in a chronic inflammatory process.

When exposed to pro-inflammatory cytokines, keratinocytes not only exhibit high proliferative activity but also start producing elevated levels of chemokines, such as CXCL1, CXCL8, CCL2 and CCL20. These cytokines contribute to the recruitment of various immune cells, such as dendritic cells, macrophages, neutrophils and Th17 cells, which are involved in the inflammatory process. Keratinocytes also generate ROS, attracting neutrophils, which will accumulate in psoriatic skin lesions [63]. There is a feedback loop in which inflammation stimulates the production of ROS and, in turn, ROS enhance the inflammatory process [64].

*a. Thioredoxin (Trx)*. The Trx complex is an antioxidant system that includes Trx, Trx reductase (TrxR) and NADPH, with a pivotal role in modulating numerous signalling pathways involved in maintaining intracellular redox homeostasis [65]. Kiafar et al. have evaluated the activity of Trx in psoriasis lesions in a small study. They identified a statistically significant negative correlation between TrxR activity and disease severity. In patients with severe forms of psoriasis, the enzyme activity was reduced [52]. The role of Trx in the pathogenesis of psoriasis is not fully elucidated, and controversies persist. On the one hand, Trx is involved in the defence against the negative effects of ROS [66], but on the other hand, the Trx/TrxR complex acts as a stimulator of angiogenesis and cell proliferation [52].

*b. Ischemia modified albumin (IMA)*. The structure of albumin is modified by ROS under conditions of hypoxia and inflammation, resulting in an altered form of the molecule called IMA, which is currently considered an important marker of oxidative stress. Several studies indicated markedly elevated levels of IMA in psoriasis patients compared to the control group. The elevated IMA levels in psoriasis patients are likely attributable to the high levels of oxidative stress observed in this population. Regarding the association between serum IMA levels and the severity of the disease, the outcomes appear to be conflicting; however, some authors consider that IMA should be regarded as a marker of disease severity [53]. The study conducted by Isik et al. suggests that patients with psoriasis who have elevated serum levels of IMA are at a higher risk of developing comorbidities [67].

*c. Monocyte-to-HDL cholesterol ratio (MHR)*. MHR represents a marker for both inflammation and oxidative stress, considering that HDL has antioxidant and anti-inflammatory action, while monocytes play a pro-inflammatory role [68]. Sirin et al. identified a positive correlation between PASI and MHR, high-sensitivity C-reactive protein and serum amyloid A [54], being the first study to evaluate MHR in psoriasis. Circulating monocytes enter the subendothelial space, where they transform into macrophages, engulfing oxidised LDL molecules and becoming foam cells capable of releasing pro-inflammatory cytokines that activate not only other monocytes but also lymphocytes and platelets [54]. These results are in accordance with those obtained by AlShamma et al. [69]. There is limited research on serum amyloid A and psoriasis; however, Dogan et al. have shown that serum amyloid A is a better indicator of inflammation than C-reactive protein in psoriasis patients [70].

**Table 3:** Hormone-related markers and disease severity.

Marker	Correlation with Disease Severity	Reference
Testosterone	negative	Allam et al. [71]
ACTH/cortisol	positive	Pietrzak et al. [72]
Prolactin	positive	Lee et al. [73]

### 3.3. Hormone-Related Markers

Hormones are molecules with a complex role in the human body, and disturbances in their levels can have effects on almost any organ [74,75,76]. Glucocorticoids have an anti-inflammatory effect by decreasing the recruitment of inflammatory cells and inhibiting T-lymphocyte function. Low levels of cortisol have been observed in patients with psoriasis. Instead, elevated levels of epinephrine and adrenocorticotropic hormones were observed. Among the effects of prolactin that favour the appearance of psoriasis plaques are the stimulation of IL-17 production by keratinocytes, recruitment of T lymphocytes at the skin level and stimulation of angiogenesis and IFN gamma production by inflammatory cells [77]. Receptors for thyroid hormones are found in the skin; therefore, thyroid activity leads to increased levels of epidermal growth factor involved in keratinocyte proliferation. Treatment with antithyroid drugs has been observed to be associated with improvement in psoriasis lesions. Considering that the skin and the endocrine system have a common embryological origin, there is a close connection between them. Hormones play an important role in the proliferation and differentiation of keratinocytes. Also, in the skin, many hormones are synthesised, including corticosteroids and sex hormones, which makes the skin considered a neuroendocrine organ [77,78,79].

*a. Testosterone*. Allam et al. emphasise the role of male hormones in psoriasis. They have shown that severe forms of psoriasis are correlated with a low serum level of testosterone. A negative correlation was identified between total testosterone and free testosterone and PASI, regardless of the patient’s age (above or below 40 years). It is known that testosterone can act as an immunosuppressive factor, down-regulating the activity of dendritic cells, interfering with the differentiation of B cells and reducing the release of pro-inflammatory cytokines [71]. Moreover, in an animal model, Schwinge et al. demonstrated that testosterone can function as an inhibitor of liver inflammation by reducing the levels of IL-17, an interleukin known to play a pivotal role in the development of psoriasis [80].

*b. Hypothalamic-pituitary-adrenal axis*. Previous studies have indicated a possible role of the hypothalamic–pituitary–adrenal axis in the pathogenesis of psoriasis. Persistent stress, which is usually encountered in psoriasis patients, can result in a dysregulation of the hypothalamic–pituitary–adrenal axis, which can promote a pro-inflammatory status [81]. Pietrzak et al. suggest that alterations in the hypothalamic–pituitary–adrenal axis may be involved in the pathogenesis of psoriasis, and a more in-depth study of the mechanisms involved could provide new insights into the processes underlying the development of psoriasis lesions. The alteration of the hypothalamic–pituitary–adrenal axis may be responsible for the release of pro-inflammatory cytokines involved in the pathogenesis of psoriasis. They found a positive correlation between morning ACTH/cortisol ratio and disease severity evaluated using the PASI score [72]. There is evidence of a correlation between salivary cortisol concentration and psoriasis severity. Additionally, a blunted salivary cortisol diurnal rhythm has been observed in psoriatic patients, suggesting a direct relationship between hypothalamic–pituitary–adrenal activity and psoriasis pathogenesis [82].

*c. Prolactin*. It is assumed that prolactin, a polypeptide hormone secreted by the anterior pituitary gland, stimulates the proliferation of keratinocytes, acts as an inductor of angiogenesis and promotes the infiltration of psoriasis lesions with Th1 cells [83]. In addition, prolactin has complex roles in regulating the immune response; it can act as a stimulator of humoral and cellular immune responses, as well as Th1 and Th17 responses. Additionally, it has been noted that prolactin can stimulate the proliferation of keratinocytes. The fact that hormones play important roles in the pathogenesis of psoriasis is supported by a recent meta-analysis that evaluates the relationship between circulating prolactin levels and the severity of psoriasis. The meta-analysis included 12 studies with a total of 446 psoriasis patients and 401 healthy subjects. Circulating prolactin levels were significantly higher among psoriasis patients, and a positive correlation with disease severity was identified [73]. Keen et al. suggest that it would be useful to perform controlled therapeutic trials of bromocriptine (a dopamine receptor agonist used in the treatment of hyperprolactinemia) in the case of patients with psoriasis, especially in the case of those with severe forms, even if the prolactin level is normal [84].

### 3.4. Cancer-Related Markers

*a. Squamous cell carcinoma antigen (SCCA)*. SCCA is a well-known marker for squamous cell carcinoma in various organs such as the head and neck, cervix, lung, etc. [85]. The serum level of SCCA correlates with the clinical stage and tumour differentiation grade. High levels of SCCA were also detected in several inflammatory disorders, such as psoriasis and atopic dermatitis [86].

The role of SCCA in psoriasis is still not well understood. Sun et al. observed a correlation between serum SCCA levels and the severity of psoriasis. Although SCCA is typically found in the cytoplasm, it has been identified in the cell nucleus in psoriasis lesions, where it inhibits apoptosis, resulting in increased keratinocyte proliferation [87]. The expression of SCCA is closely related to the activity of Th17 and Th22 cells. The inflammatory reaction that arises due to the cytokine release triggered by the activation of Th17 and Th22 cells plays a role in the development of psoriasis. Additionally, the cytokines can lead to elevated SCCA expression in tissues. Ghonemy et al. demonstrated, in a recent study, that SCCA expression is higher in psoriasis lesions compared to healthy skin and is positively correlated with the abundance of inflammatory cells in the dermis and disease severity. These findings suggest that SCCA could be considered both a marker of disease severity and an important tool for assessing inflammation [88].

## 4. Discussion

Psoriasis has been described as an inflammatory disease mediated by T cells, but in fact, numerous cells are involved in its pathogenesis, including keratinocytes, neutrophils, dendritic cells, mast cells and endothelial cells. Macrophages and NK cells are responsible for the occurrence of histopathological changes, such as hyperkeratosis with parakeratosis, enlargement of dermal vessels, the presence of Munro’s microabscesses and the increase in size of dermal papillae. Thus, it can be stated that most of the skin cells play a role in the pathogenesis of psoriasis. Keratinocytes, dendritic cells and T cells release a variety of cytokines (IL-8, IL-17, IL2-2, IL-23, TNF alpha, etc.) that mediate the complex intercellular interaction and lead to the accelerated proliferation of keratinocytes and abnormal differentiation, angiogenesis and pro-inflammatory response, additional inflammatory cells being recruited and activated, which will perpetuate an inflammatory reaction [89,90]. Thus, the defining characteristic of psoriasis is persistent inflammation, orchestrated by a multitude of cells and molecules, some of which are better understood than others.

In this review, we have identified a group of molecules with an immunomodulatory effect (Table 1) that can be measured in the serum of patients with psoriasis, and their levels correlate with the severity of the disease. This fact demonstrates their role in the pathogenesis of psoriasis and opens new perspectives on the modulation of the inflammatory response in these patients. Thus, for example, current studies have shown changes in the serum levels of YKL-40 after narrow-band UVB therapy or of elafin after cyclosporine treatment [15,17]. Moreover, these molecules seem to be involved in the comorbidities associated with psoriasis; therefore, a negative correlation was observed between HDL cholesterol levels and FGL1, indicating a possible role of FGL1 in psoriasis forms associated with lipid metabolism disorders [38] or YKL-40 may be involved in endothelial dysfunction [17].

The measurement of haematological parameters is an easy and inexpensive method to perform in everyday medical practice, but the data from recent years regarding the correlation of these parameters with the severity of psoriasis are not convincing. Further studies are needed.

In the analysed studies, we identified oxidative stress makers that can be correlated with the severity of the disease (Trx, IMA, MHR). Increased levels of pro-oxidants and decreased antioxidant capacity suggest a role for antioxidant therapies in psoriasis. Antioxidant systems play a defining role in the defence against ROS. Superoxide dismutase and catalase are the most studied antioxidants in patients with psoriasis. Many studies indicate low serum levels of these enzymes in patients with psoriasis, but there are also studies that show elevated serum levels. Antioxidant therapies could potentially represent a useful therapeutic approach for these patients, but further studies are needed [62]. A recent analysis of the available data on a diet rich in antioxidants in psoriasis indicates that a diet rich in vitamins C, A, D and E, as well as polyphenolic compounds found in tea and coffee, can have beneficial effects [91].

The skin harbours a complex endocrine system, playing a pivotal role in numerous pathological conditions. There is a close link between the pathogenic processes involved in psoriasis and hormonal status. It has been shown that the levels of corticotropin-releasing hormone (CRH) in the skin of patients with psoriasis are higher compared to healthy skin. TNF-α and IFN cytokines involved in psoriasis pathogenesis increase the levels of CRH as well as of adrenocorticotropic hormone (ACTH) and cortisol, which indicate the influence of inflammation on hormonal status in these patients [92]. A hormone status evaluation may be useful in patients with psoriasis for better management of the disease [77]. The results of our review indicate a link between sex hormones, prolactin and the hypothalamic–pituitary–adrenal axis with psoriasis severity and their modulation could improve psoriasis course.

It is necessary to study novel drugs that target key molecules involved in the pathogenesis of psoriasis, which can represent the basis for the implementation of new regimens for the treatment of psoriasis, with fewer drug interactions and better patient quality of life [93].

This review gathers data regarding numerous markers studied in recent years that could provide data about the severity of psoriasis; however, it is limited to four categories (markers related to inflammation, oxidative stress, hormones and cancer-related markers). In addition, it should be mentioned that few studies are currently available, and further research is needed.

## 5. Conclusions

Psoriasis is a multifactorial disease with a complex pathogenesis that is incompletely elucidated. Over time, various markers related to the processes involved in its pathogenesis have been investigated. Current evidence shows that markers related to inflammation, oxidative stress, hormones and cancer-related markers may be useful in the evaluation of psoriasis severity; however, further studies are needed to determine the significance of these biomarkers in medical practice.

## Figures and Tables

**Table 1:** The link between inflammation-related markers and disease severity.

Marker	Correlation with Disease Severity	Reference
Immunomodulatory molecules		
Neopterin	positive	Kemeriz et al. [14]
Elafin	positive	Elgharib et al. [15]
FGL1	negative	Sun et al. [16]
YKL-40	positive	Khashaba et al. [17]
GDF-15	positive	Akbari et al. [18]
Lipocalin-2	positive	Nguyen et al. [19]
SLURP1	positive	Assaf et al. [20]
Hematological markers		
Platelet count	negative	Nageen et al. [21]
MPW	no correlation	Nageen et al. [21]
RDW	no correlation	Nageen et al. [21]
Inflammatory cells and costimulatory molecules		
Tph cells	positive	Liu et al. [22]
CD86^+^ monocytes	positive	Nguyen et al. [23]
Membrane CTLA4 Soluble CTLA4	positive no correlation	Liu et al. [24]

FGL1—fibrinogen-like protein I, GDF-15—growth/differentiation factor-15, SLURP1—the secreted Ly6/urokinase-type plasminogen activator receptor-related protein-1, RDW—red cell distribution width, Tph cells—peripheral helper T cells, CTLA4—cytotoxic T-lymphocyte associated protein 4.
